# Functional specificity of the locus coeruleus-norepinephrine system in the attentional networks

**DOI:** 10.3389/fnbeh.2013.00201

**Published:** 2013-12-12

**Authors:** Ettore Ambrosini, Roberta Vastano, Maria Montefinese, Marco Ciavarro

**Affiliations:** ^1^Laboratory of Neuropsychology and Cognitive Neuroscience, Department of Neuroscience and Imaging, University G. d'AnnunzioChieti, Italy; ^2^Institute for Advanced Biomedical Technologies - ITAB, Foundation G. d'AnnunzioChieti, Italy; ^3^Department of Psychological, Humanistic and Territorial Sciences, University G. d'AnnunzioChieti, Italy; ^4^“Neurone” Foundation for Research in Neuropsychobiology and Clinical NeurosciencesRome, Italy

**Keywords:** attention, pupil response, norepinephrine, locus coeruleus, adaptive gain theory

Attention is one of the oldest and most pivotal themes in cognitive science. A current and widely accepted theory holds that anatomically-distinct, hierarchically-organized networks, each responsible for specific components of the attention process (i.e., alerting, orienting, and executive control), constitute the attention system (Posner and Petersen, 1990). Alerting prepares the system for fast reactions to upcoming stimuli, orienting allows prioritizing sensory input by selecting a spatial location or modality while executive control involves error monitoring and conflict resolving. The Attention Network Test (ANT), combining cued detection (Posner, 1980) with a flanker-type paradigm (Eriksen and Eriksen, 1974), has recently been developed to behaviorally assess processing efficiency within these networks (Fan et al., 2002).

Geva et al. (2013) recently used the ANT and psychosensory pupil dilation (PD) responses to examine the temporo-spatial attributes of concurrent locus coeruleus-norepinephrinergic (LC–NE) activity, notoriously involved in alerting (Rajkowski et al., 1994), and hypothesized by them to affect orienting and executive control networks as well. Their analyses identified an early and a late PD peak, which they called Pa and Pe, respectively. The reported results seem to suggest that Pa characterizes both alerting and orienting processes, since the temporally informative (alerting) double-cue evoked larger Pa amplitude compared to the no-cue condition, whereas the temporally-and-spatially informative cue additionally accelerated Pa activation compared to the alerting cue. On the contrary, Pe characterizes executive control processes, as its amplitude was larger in the incongruent than both neutral and congruent conditions. Based on these results, the authors concluded that PD responses seem to be “evoked in each attention network in a construct-specific manner.”

A first interesting aspect of Geva et al.'s paper relates to the investigation of the putative interaction among the different attention networks, and in particular between the alerting and orienting components, a somewhat controversial topic that has recently received great interest. In fact, while Callejas et al. (2004) showed that alerting accelerated orienting, Fuentes and Campoy (2008) provided evidence supporting the competing enhancing hypothesis. On the contrary, Geva et al. argued for an (opposite) effect of orienting on alerting processes, with the former accelerating the latter response. Unluckily, they did not provide clear explanations of this peculiar effect, nor result details needed to appreciate it, both for the pupillary and behavioral results. A factor that likely determined this shortcoming is that, as pointed out by Callejas et al. (2004), the original ANT paradigm used by Geva et al. does not allow measuring the effect of each network on the other two independently, and especially the alerting-orienting interaction, since both these processes are investigated using different levels of the same variable (i.e., the four cue conditions). Another likely reason is linked to the vagueness in defining how the early PD response relates to attention processes. Indeed, Geva et al. defined the Pa as the evoked PD response due to the phasic LC–NE activation (Aston-Jones and Cohen, 2005) linked to alertness. At the same time, however, the PD response “evoked in (the orienting network) in a construct-specific manner” was the same Pa evoked in the alerting network, and the authors claimed that it represents “the recruitment of autonomic resources required for alerting and covert attention shifts.” However, it is unclear how the early PD response could be the neurophysiological correlate of both these attention processes. It would have been useful to investigate the different components reflecting distinct, independent cognitive mechanisms by analyzing the pupillary response with a principal component analysis (e.g., Nuthmann and Van Der Meer, 2005). Their manual procedure used to identify the PD peaks does not allow this to be done in a clear and unquestionable manner.

A second aspect of Geva et al.'s work that deserves particular attention is related to their attempt to extend the Aston-Jones and Cohen's (2005) adaptive gain theory (AGT) “to encompass all three attention networks.” The AGT relies on neurophysiological findings revealing two modes of LC activity (phasic and tonic) and, rather than addressing arousal *per se* as in earlier theories of LC–NE activity (Aston-Jones et al., 1991), it specifies a role for the different modes of LC–NE system activity in optimizing behavioral performance (Aston-Jones and Cohen, 2005). Regrettably, Geva et al.'s references to AGT can be questionable. First, according to them, only Pe would reflect the exploitation LC mode, as “the LC–NE system is activated in the phasic mode during more demanding tasks.” However, it is unclear why the phasic mode should be activated even in the low-demanding congruent condition. Moreover, according to the AGT, the phasic (exploitation) LC–NE mode, characterized by phasic, event-locked responses to task-relevant stimuli and related to high levels of task performance, is driven by the outcome of decision processes associated with high task-related utility encoded in frontal structures. Second, Geva et al. claimed that Pa reflects the exploration LC mode, elicited by “a non-specific alerting cue or the absence of a specific cue.” However, the AGT relates the tonic (exploration) LC–NE mode, characterized by both high tonic activity and a relative lack of phasic responses to task-relevant events, to poor task performance (task disengagement), increased distractibility, and higher responsiveness to all events. Nevertheless, Geva et al. did not assess neither its relation with participants' performance, nor baseline pupil diameter. Moreover, AGT holds that the exploration LC–NE mode is driven by low long-term task-related utility encoded in frontal (orbitofrontal and anterior cingulate) cortices, and thus its activation is not compatible with a short-lived but task-relevant event such us the presentation of a (albeit non-specific) cue.

In summary, Geva et al. (2013) provide evidence for the role of the LC–NE system in the three attention processes, but both the exact mechanisms governing its involvement and whether and how the attention networks interact remain unclear. Furthermore, their conclusions are not easily reconciled with the AGT. Thus, additional research is required to fully understand the temporo-spatial attributes of the putative construct-specific influence of the LC–NE system in the alerting, orienting, and executive control attention processes.
